# College and Hospital Notes

**Published:** 1908-12

**Authors:** 


### COLLEGE AND HOSPITAL NOTES

---

The Hospital for Contagious Diseases has made a start in the action by the board of aldermen, in passing a resolution asking for a $250,000 enabling act. This first step may be a long way from the conclusion of the effort, but it is at least a beginning. Let the work go on.

---

Dr. George Dock of the Medical Department, Tulane University, is delivering a course of special lectures which began October 28, 1908. The remainder of the schedule includes:

December 16, VII.—Exophthalmic Goitre (Concluded): Complications; varieties; prognosis; diagnosis; treatment. Athyreosis and Hypotyreosis: (Cretinism and myxedema): Historical; endemic; etiology; pathology and pathologic anatomy; clinical features; treatment.

January 6, VIII.—Sporadic Cretinism: Etiology; clinical features; prognosis; diagnosis; treatment. Myxedema: Etiology pathologic anatomy; symptoms; varieties; diagnosis; course and prognosis; treatment.

January 13, IX.—The Pituitary Body: Physiology; pathologic anatomy. Acromegaly: Historical; etiology; morbid anatomy; clinical features; prognosis; diagnosis; treatment. Progeria.

January 20, X.—Osteomalacia. Historical; etiology; morbid anatomy; clinical course; diagnosis; prophylaxis; treatment.

